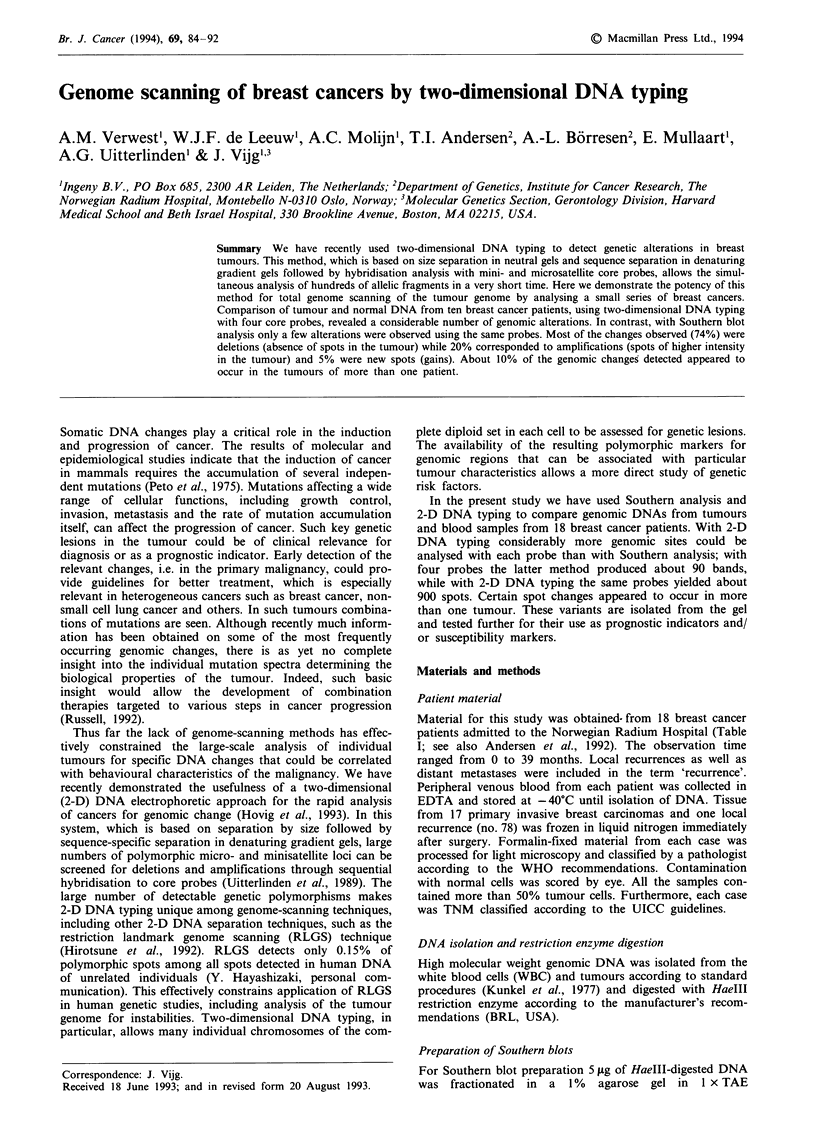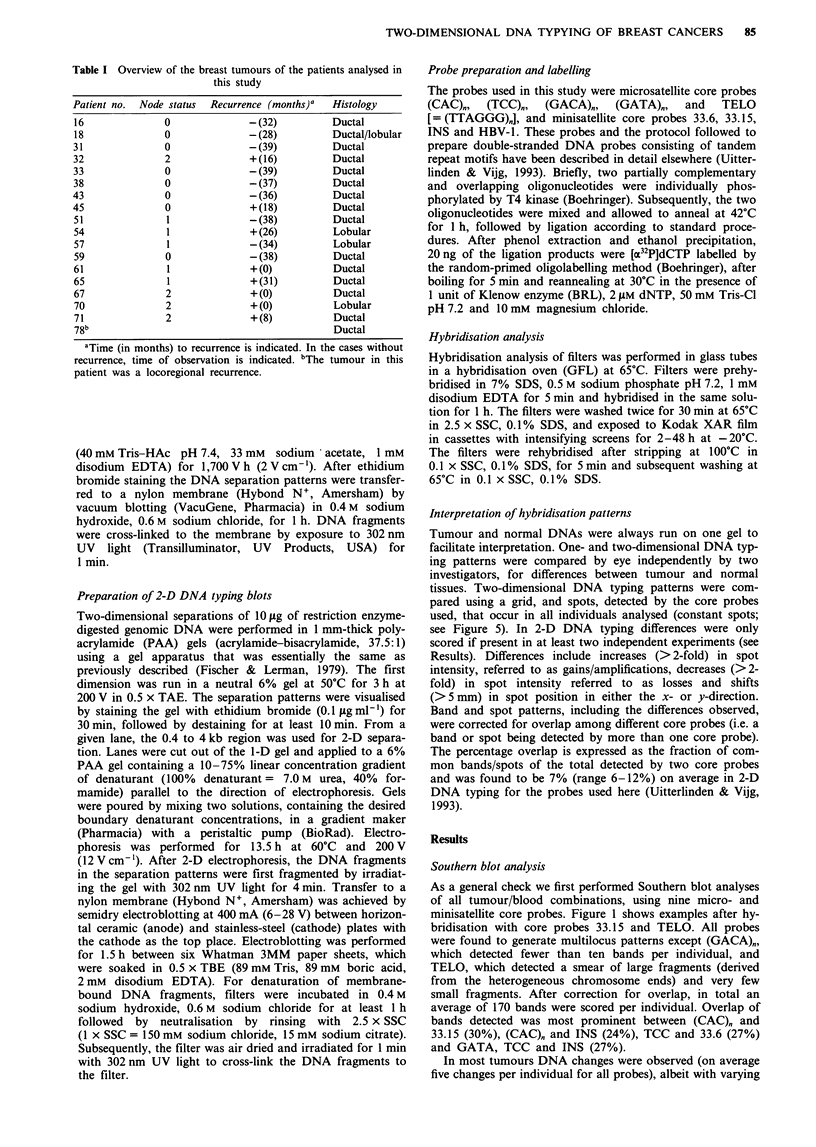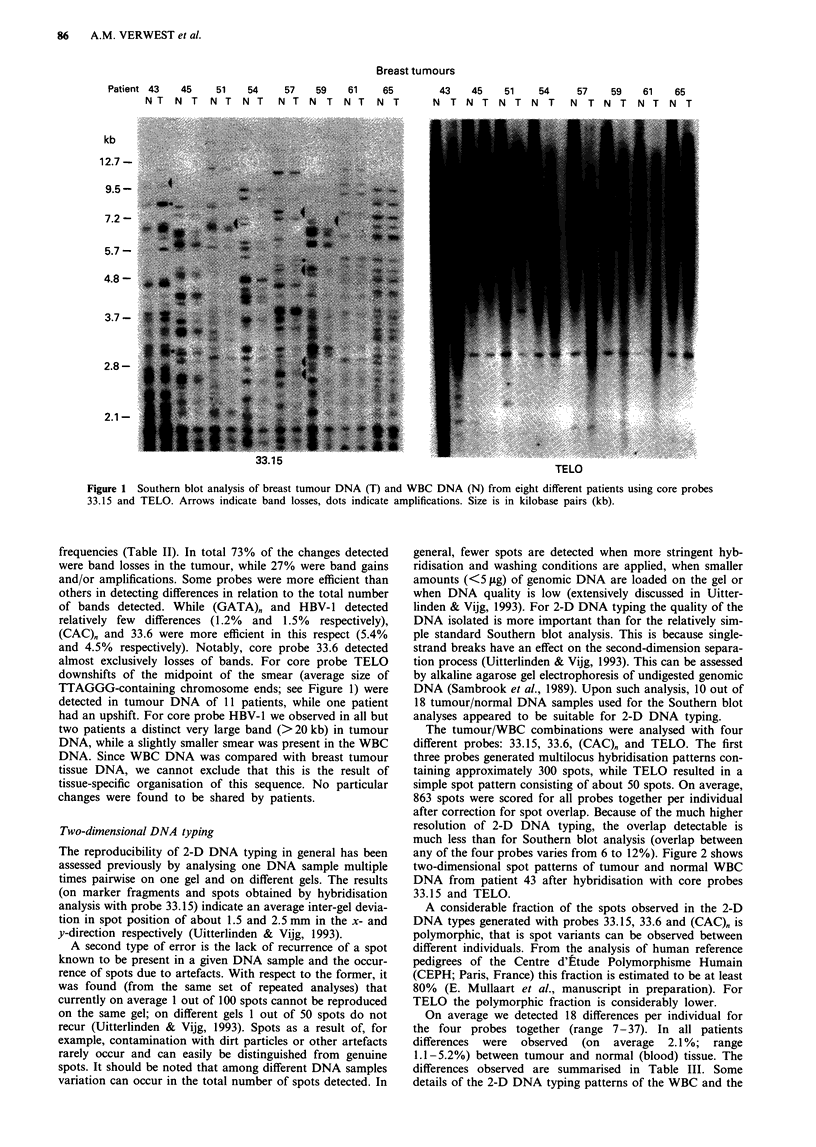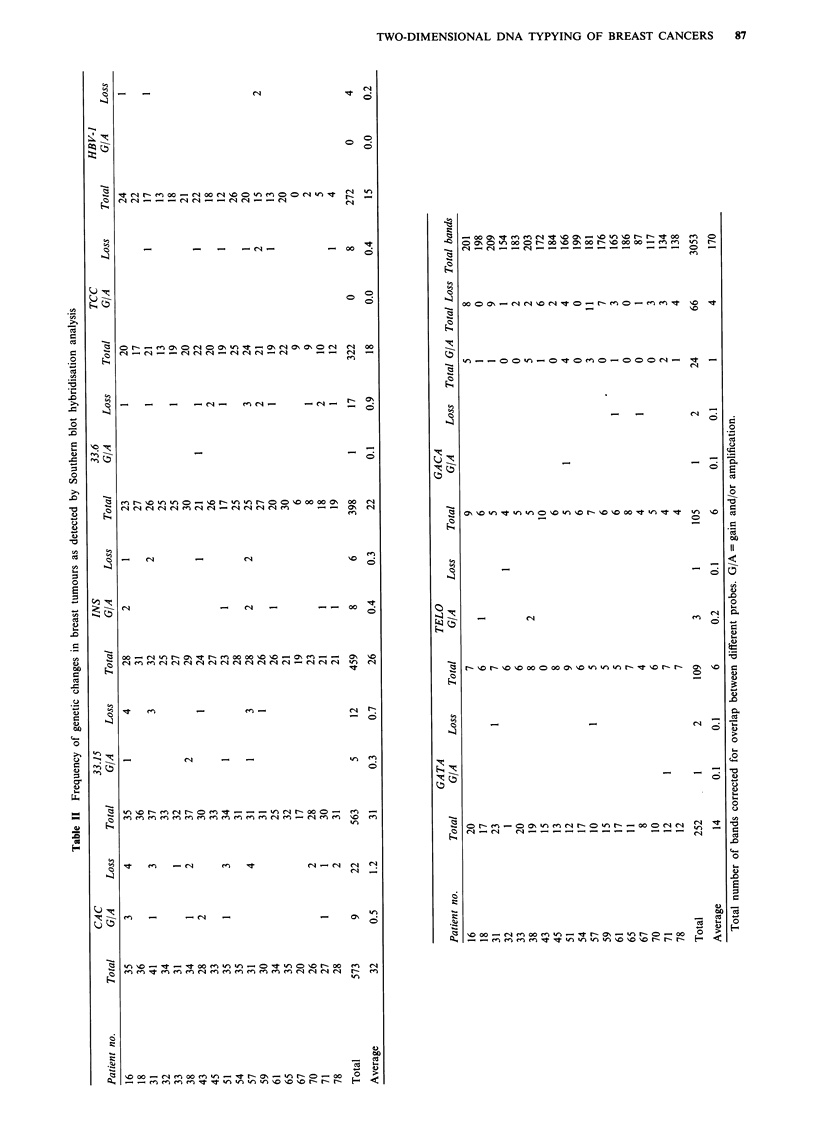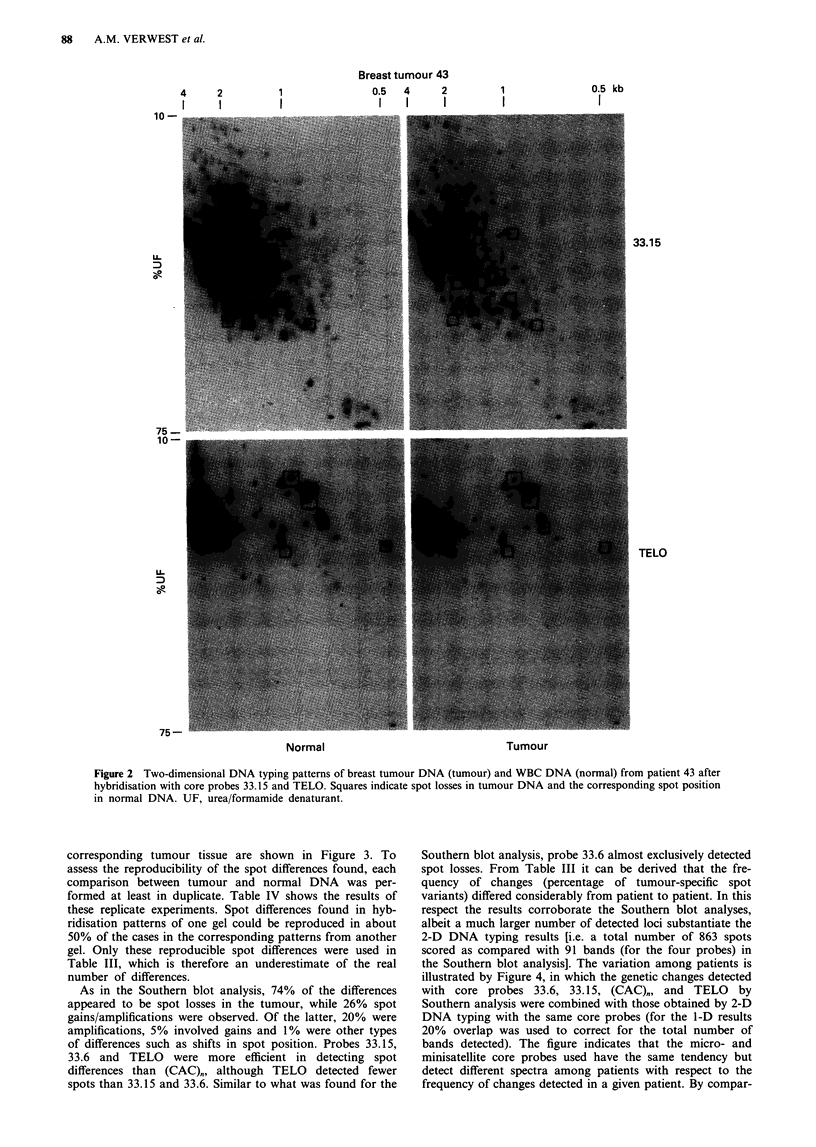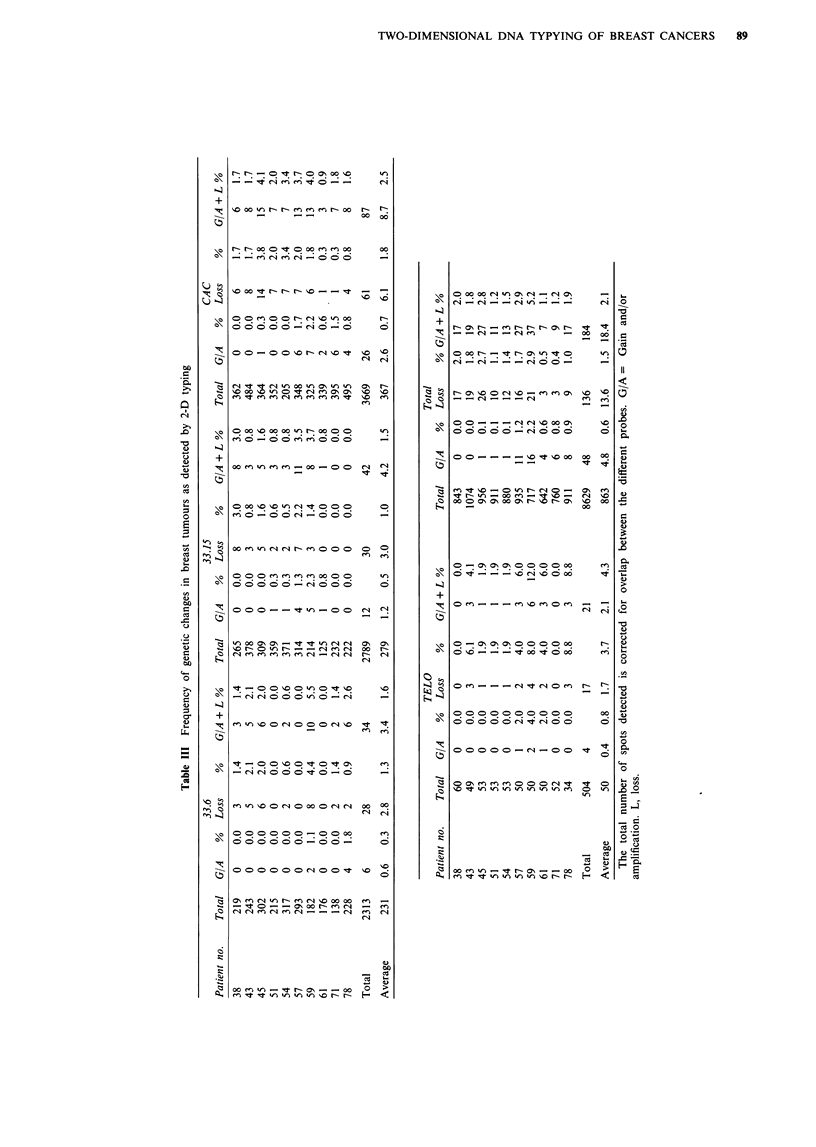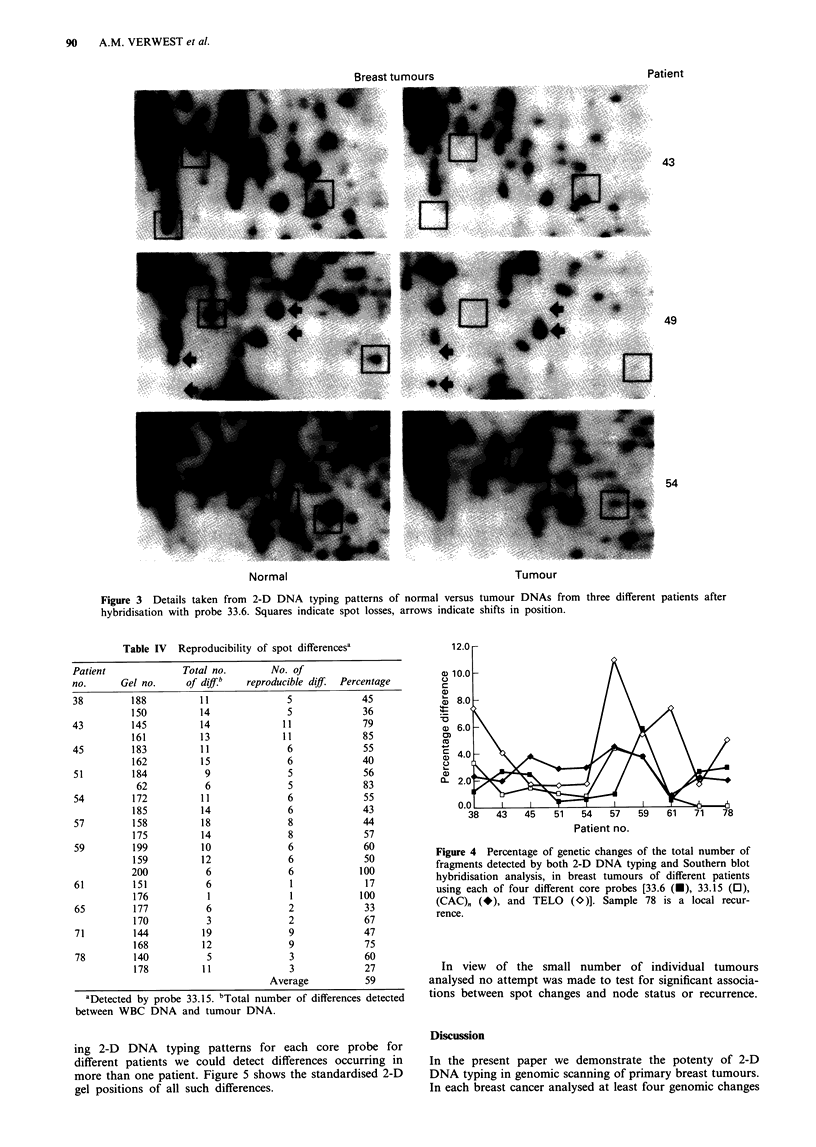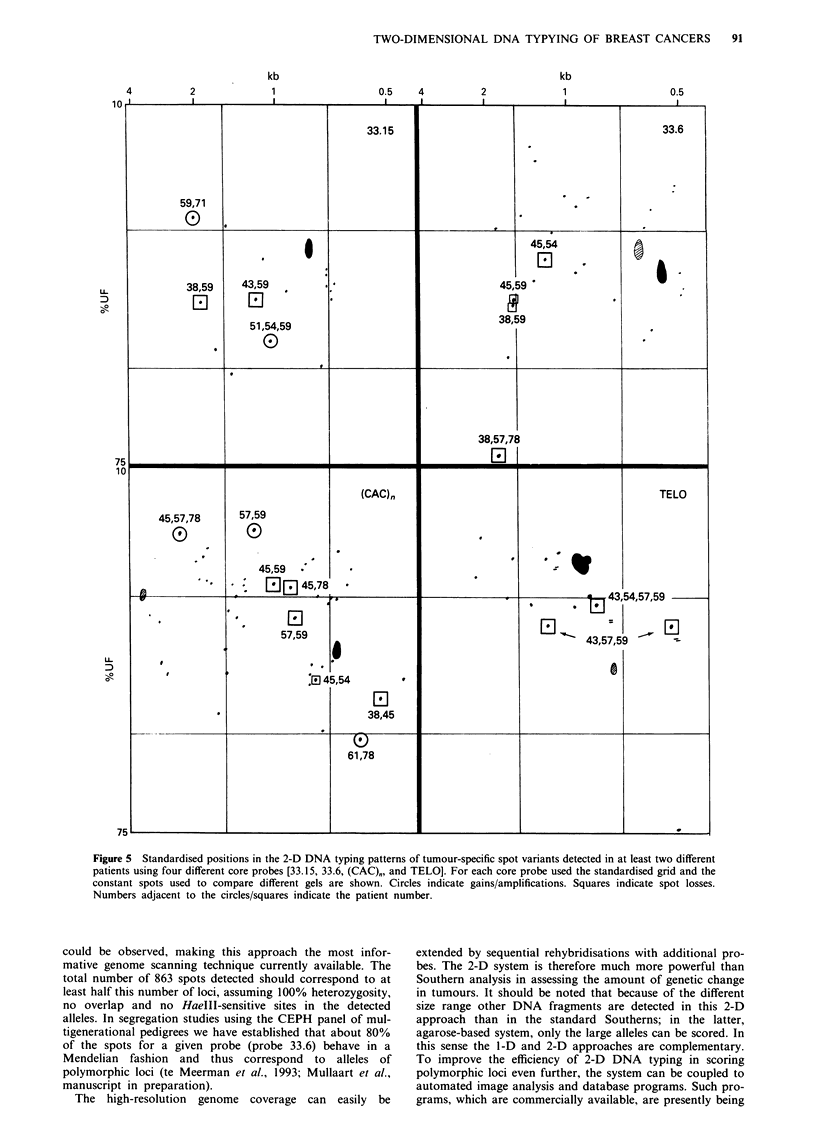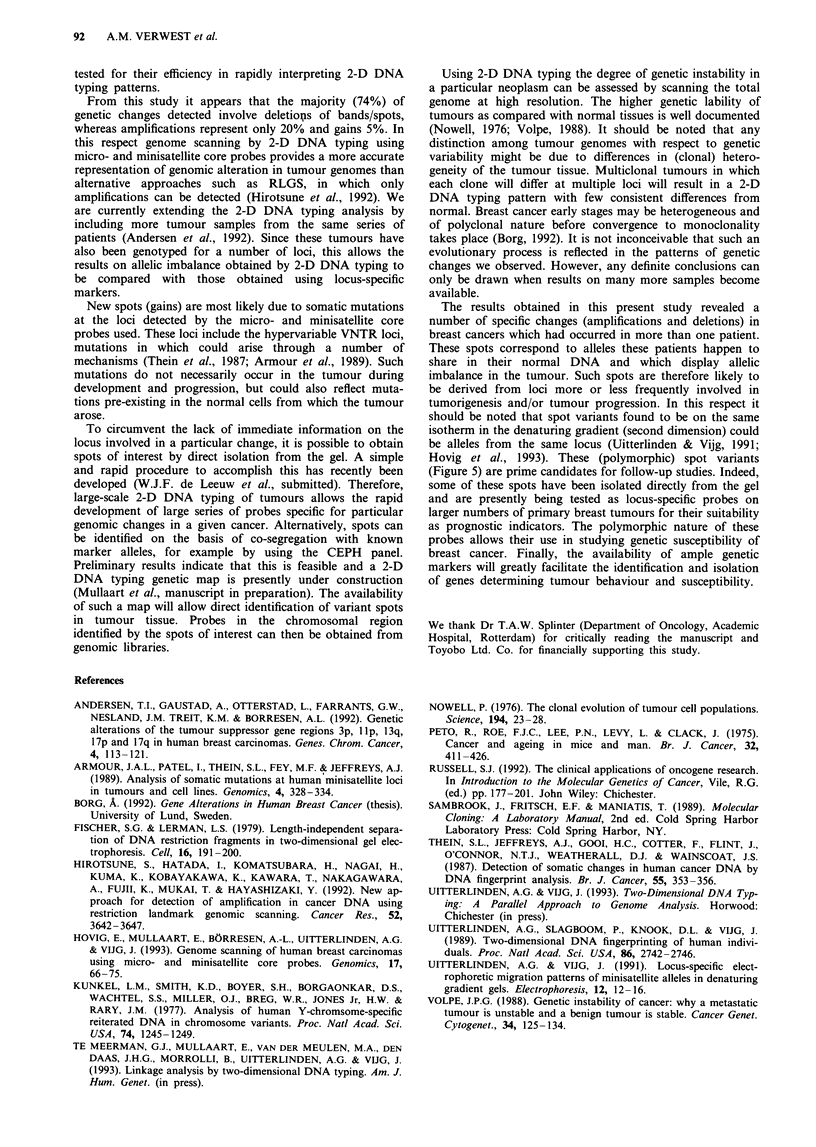# Genome scanning of breast cancers by two-dimensional DNA typing.

**DOI:** 10.1038/bjc.1994.13

**Published:** 1994-01

**Authors:** A. M. Verwest, W. J. de Leeuw, A. C. Molijn, T. I. Andersen, A. L. Börresen, E. Mullaart, A. G. Uitterlinden, J. Vijg

**Affiliations:** Ingeny B.V., Leiden, The Netherlands.

## Abstract

**Images:**


Br. J. Cancer (1994), 69, 84-92 © Macmillan Press Ltd., 1994

### Genome scanning of breast cancers by two-dimensional DNA typing

A.M. Verwest[1], W.J.F. de Leeuw[1], A.C. Molijn[1], T.I. Andersen[2], A.-L. Börresen[2], E. Mullaart[1], A.G. Uitterlinden[1] & J. Vijg[1,3]

[1]Ingeny B.V., PO Box 685, 2300 AR Leiden, The Netherlands; [2]Department of Genetics, Institute for Cancer Research, The Norwegian Radium Hospital, Montebello N-0310 Oslo, Norway; [3]Molecular Genetics Section, Gerontology Division, Harvard Medical School and Beth Israel Hospital, 330 Brookline Avenue, Boston, MA 02215, USA.

**Summary** We have recently used two-dimensional DNA typing to detect genetic alterations in breast tumours. This method, which is based on size separation in neutral gels and sequence separation in denaturing gradient gels followed by hybridisation analysis with mini- and microsatellite core probes, allows the simultaneous analysis of hundreds of allelic fragments in a very short time. Here we demonstrate the potency of this method for total genome scanning of the tumour genome by analysing a small series of breast cancers. Comparison of tumour and normal DNA from ten breast cancer patients, using two-dimensional DNA typing with four core probes, revealed a considerable number of genomic alterations. In contrast, with Southern blot analysis only a few alterations were observed using the same probes. Most of the changes observed (74%) were deletions (absence of spots in the tumour) while 20% corresponded to amplifications (spots of higher intensity in the tumour) and 5% were new spots (gains). About 10% of the genomic changes detected appeared to occur in the tumours of more than one patient.

Somatic DNA changes play a critical role in the induction and progression of cancer. The results of molecular and epidemiological studies indicate that the induction of cancer in mammals requires the accumulation of several independent mutations (Peto *et al.*, 1975). Mutations affecting a wide range of cellular functions, including growth control, invasion, metastasis and the rate of mutation accumulation itself, can affect the progression of cancer. Such key genetic lesions in the tumour could be of clinical relevance for diagnosis or as a prognostic indicator. Early detection of the relevant changes, i.e. in the primary malignancy, could provide guidelines for better treatment, which is especially relevant in heterogeneous cancers such as breast cancer, non-small cell lung cancer and others. In such tumours combinations of mutations are seen. Although recently much information has been obtained on some of the most frequently occurring genomic changes, there is as yet no complete insight into the individual mutation spectra determining the biological properties of the tumour. Indeed, such basic insight would allow the development of combination therapies targeted to various steps in cancer progression (Russell, 1992).

Thus far the lack of genome-scanning methods has effectively constrained the large-scale analysis of individual tumours for specific DNA changes that could be correlated with behavioural characteristics of the malignancy. We have recently demonstrated the usefulness of a two-dimensional (2-D) DNA electrophoretic approach for the rapid analysis of cancers for genomic change (Hovig *et al.*, 1993). In this system, which is based on separation by size followed by sequence-specific separation in denaturing gradient gels, large numbers of polymorphic micro- and minisatellite loci can be screened for deletions and amplifications through sequential hybridisation to core probes (Uitterlinden *et al.*, 1989). The large number of detectable genetic polymorphisms makes 2-D DNA typing unique among genome-scanning techniques, including other 2-D DNA separation techniques, such as the restriction landmark genome scanning (RLGS) technique (Hirotsune *et al.*, 1992). RLGS detects only 0.15% of polymorphic spots among all spots detected in human DNA of unrelated individuals (Y. Hayashizaki, personal communication). This effectively constrains application of RLGS in human genetic studies, including analysis of the tumour genome for instabilities. Two-dimensional DNA typing, in particular, allows many individual chromosomes of the complete diploid set in each cell to be assessed for genetic lesions. The availability of the resulting polymorphic markers for genomic regions that can be associated with particular tumour characteristics allows a more direct study of genetic risk factors.

In the present study we have used Southern analysis and 2-D DNA typing to compare genomic DNAs from tumours and blood samples from 18 breast cancer patients. With 2-D DNA typing considerably more genomic sites could be analysed with each probe than with Southern analysis; with four probes the latter method produced about 90 bands, while with 2-D DNA typing the same probes yielded about 900 spots. Certain spot changes appeared to occur in more than one tumour. These variants are isolated from the gel and tested further for their use as prognostic indicators and/or susceptibility markers.

### Materials and methods

### Patient material

Material for this study was obtained from 18 breast cancer patients admitted to the Norwegian Radium Hospital (Table I; see also Andersen *et al.*, 1992). The observation time ranged from 0 to 39 months. Local recurrences as well as distant metastases were included in the term 'recurrence'. Peripheral venous blood from each patient was collected in EDTA and stored at −40°C until isolation of DNA. Tissue from 17 primary invasive breast carcinomas and one local recurrence (no. 78) was frozen in liquid nitrogen immediately after surgery. Formalin-fixed material from each case was processed for light microscopy and classified by a pathologist according to the WHO recommendations. Contamination with normal cells was scored by eye. All the samples contained more than 50% tumour cells. Furthermore, each case was TNM classified according to the UICC guidelines.

### DNA isolation and restriction enzyme digestion

High molecular weight genomic DNA was isolated from the white blood cells (WBC) and tumours according to standard procedures (Kunkel *et al.*, 1977) and digested with *Hae*III restriction enzyme according to the manufacturer's recommendations (BRL, USA).

### Preparation of Southern blots

For Southern blot preparation 5 µg of *Hae*III-digested DNA was fractionated in a 1% agarose gel in 1 × TAE

Correspondence: J. Vijg.
Received 18 June 1993; and in revised form 20 August 1993.

**Table I** Overview of the breast tumours of the patients analysed in this study

| *Patient no.* | *Node status* | *Recurrence (months)*[a] | *Histology* |
|---|---|---|---|
| 16 | 0 | −(32) | Ductal |
| 18 | 0 | −(28) | Ductal/lobular |
| 31 | 0 | −(39) | Ductal |
| 32 | 2 | +(16) | Ductal |
| 33 | 0 | −(39) | Ductal |
| 38 | 0 | −(37) | Ductal |
| 43 | 0 | −(36) | Ductal |
| 45 | 0 | +(18) | Ductal |
| 51 | 1 | −(38) | Ductal |
| 54 | 1 | +(26) | Lobular |
| 57 | 1 | −(34) | Lobular |
| 59 | 0 | −(38) | Ductal |
| 61 | 1 | +(0) | Ductal |
| 65 | 1 | +(31) | Ductal |
| 67 | 2 | +(0) | Ductal |
| 70 | 2 | +(0) | Lobular |
| 71 | 2 | +(8) | Ductal |
| 78[b] | | | Ductal |

[a]Time (in months) to recurrence is indicated. In the cases without recurrence, time of observation is indicated. [b]The tumour in this patient was a locoregional recurrence.

(40 mM Tris–HAc pH 7.4, 33 mM sodium acetate, 1 mM disodium EDTA) for 1,700 V h (2 V $cm^{-1}$). After ethidium bromide staining the DNA separation patterns were transferred to a nylon membrane (Hybond $N^+$, Amersham) by vacuum blotting (VacuGene, Pharmacia) in 0.4 M sodium hydroxide, 0.6 M sodium chloride, for 1 h. DNA fragments were cross-linked to the membrane by exposure to 302 nm UV light (Transilluminator, UV Products, USA) for 1 min.

*Preparation of 2-D DNA typing blots*

Two-dimensional separations of 10 µg of restriction enzyme-digested genomic DNA were performed in 1 mm-thick polyacrylamide (PAA) gels (acrylamide–bisacrylamide, 37.5:1) using a gel apparatus that was essentially the same as previously described (Fischer & Lerman, 1979). The first dimension was run in a neutral 6% gel at 50°C for 3 h at 200 V in 0.5 × TAE. The separation patterns were visualised by staining the gel with ethidium bromide (0.1 µg $ml^{-1}$) for 30 min, followed by destaining for at least 10 min. From a given lane, the 0.4 to 4 kb region was used for 2-D separation. Lanes were cut out of the 1-D gel and applied to a 6% PAA gel containing a 10–75% linear concentration gradient of denaturant (100% denaturant = 7.0 M urea, 40% formamide) parallel to the direction of electrophoresis. Gels were poured by mixing two solutions, containing the desired boundary denaturant concentrations, in a gradient maker (Pharmacia) with a peristaltic pump (BioRad). Electrophoresis was performed for 13.5 h at 60°C and 200 V (12 V $cm^{-1}$). After 2-D electrophoresis, the DNA fragments in the separation patterns were first fragmented by irradiating the gel with 302 nm UV light for 4 min. Transfer to a nylon membrane (Hybond $N^+$, Amersham) was achieved by semidry electroblotting at 400 mA (6–28 V) between horizontal ceramic (anode) and stainless-steel (cathode) plates with the cathode as the top place. Electroblotting was performed for 1.5 h between six Whatman 3MM paper sheets, which were soaked in 0.5 × TBE (89 mM Tris, 89 mM boric acid, 2 mM disodium EDTA). For denaturation of membrane-bound DNA fragments, filters were incubated in 0.4 M sodium hydroxide, 0.6 M sodium chloride for at least 1 h followed by neutralisation by rinsing with 2.5 × SSC (1 × SSC = 150 mM sodium chloride, 15 mM sodium citrate). Subsequently, the filter was air dried and irradiated for 1 min with 302 nm UV light to cross-link the DNA fragments to the filter.

*Probe preparation and labelling*

The probes used in this study were microsatellite core probes $(CAC)_n$, $(TCC)_n$, $(GACA)_n$, $(GATA)_n$, and TELO [= $(TTAGGG)_n$], and minisatellite core probes 33.6, 33.15, INS and HBV-1. These probes and the protocol followed to prepare double-stranded DNA probes consisting of tandem repeat motifs have been described in detail elsewhere (Uitterlinden & Vijg, 1993). Briefly, two partially complementary and overlapping oligonucleotides were individually phosphorylated by T4 kinase (Boehringer). Subsequently, the two oligonucleotides were mixed and allowed to anneal at 42°C for 1 h, followed by ligation according to standard procedures. After phenol extraction and ethanol precipitation, 20 ng of the ligation products were [$\alpha^{32}P$]dCTP labelled by the random-primed oligolabelling method (Boehringer), after boiling for 5 min and reannealing at 30°C in the presence of 1 unit of Klenow enzyme (BRL), 2 µM dNTP, 50 mM Tris-Cl pH 7.2 and 10 mM magnesium chloride.

*Hybridisation analysis*

Hybridisation analysis of filters was performed in glass tubes in a hybridisation oven (GFL) at 65°C. Filters were prehybridised in 7% SDS, 0.5 M sodium phosphate pH 7.2, 1 mM disodium EDTA for 5 min and hybridised in the same solution for 1 h. The filters were washed twice for 30 min at 65°C in 2.5 × SSC, 0.1% SDS, and exposed to Kodak XAR film in cassettes with intensifying screens for 2–48 h at −20°C. The filters were rehybridised after stripping at 100°C in 0.1 × SSC, 0.1% SDS, for 5 min and subsequent washing at 65°C in 0.1 × SSC, 0.1% SDS.

*Interpretation of hybridisation patterns*

Tumour and normal DNAs were always run on one gel to facilitate interpretation. One- and two-dimensional DNA typing patterns were compared by eye independently by two investigators, for differences between tumour and normal tissues. Two-dimensional DNA typing patterns were compared using a grid, and spots, detected by the core probes used, that occur in all individuals analysed (constant spots; see Figure 5). In 2-D DNA typing differences were only scored if present in at least two independent experiments (see Results). Differences include increases (>2-fold) in spot intensity, referred to as gains/amplifications, decreases (>2-fold) in spot intensity referred to as losses and shifts (>5 mm) in spot position in either the *x*- or *y*-direction. Band and spot patterns, including the differences observed, were corrected for overlap among different core probes (i.e. a band or spot being detected by more than one core probe). The percentage overlap is expressed as the fraction of common bands/spots of the total detected by two core probes and was found to be 7% (range 6–12%) on average in 2-D DNA typing for the probes used here (Uitterlinden & Vijg, 1993).

### Results

*Southern blot analysis*

As a general check we first performed Southern blot analyses of all tumour/blood combinations, using nine micro- and minisatellite core probes. Figure 1 shows examples after hybridisation with core probes 33.15 and TELO. All probes were found to generate multilocus patterns except $(GACA)_n$, which detected fewer than ten bands per individual, and TELO, which detected a smear of large fragments (derived from the heterogeneous chromosome ends) and very few small fragments. After correction for overlap, in total an average of 170 bands were scored per individual. Overlap of bands detected was most prominent between $(CAC)_n$ and 33.15 (30%), $(CAC)_n$ and INS (24%), TCC and 33.6 (27%) and GATA, TCC and INS (27%).

In most tumours DNA changes were observed (on average five changes per individual for all probes), albeit with varying

**Figure 1** Southern blot analysis of breast tumour DNA (T) and WBC DNA (N) from eight different patients using core probes 33.15 and TELO. Arrows indicate band losses, dots indicate amplifications. Size is in kilobase pairs (kb).

frequencies (Table II). In total 73% of the changes detected were band losses in the tumour, while 27% were band gains and/or amplifications. Some probes were more efficient than others in detecting differences in relation to the total number of bands detected. While $(GATA)_n$ and HBV-1 detected relatively few differences (1.2% and 1.5% respectively), $(CAC)_n$ and 33.6 were more efficient in this respect (5.4% and 4.5% respectively). Notably, core probe 33.6 detected almost exclusively losses of bands. For core probe TELO downshifts of the midpoint of the smear (average size of TTAGGG-containing chromosome ends; see Figure 1) were detected in tumour DNA of 11 patients, while one patient had an upshift. For core probe HBV-1 we observed in all but two patients a distinct very large band (>20 kb) in tumour DNA, while a slightly smaller smear was present in the WBC DNA. Since WBC DNA was compared with breast tumour tissue DNA, we cannot exclude that this is the result of tissue-specific organisation of this sequence. No particular changes were found to be shared by patients.

*Two-dimensional DNA typing*

The reproducibility of 2-D DNA typing in general has been assessed previously by analysing one DNA sample multiple times pairwise on one gel and on different gels. The results (on marker fragments and spots obtained by hybridisation analysis with probe 33.15) indicate an average inter-gel deviation in spot position of about 1.5 and 2.5 mm in the *x*- and *y*-direction respectively (Uitterlinden & Vijg, 1993).

A second type of error is the lack of recurrence of a spot known to be present in a given DNA sample and the occurrence of spots due to artefacts. With respect to the former, it was found (from the same set of repeated analyses) that currently on average 1 out of 100 spots cannot be reproduced on the same gel; on different gels 1 out of 50 spots do not recur (Uitterlinden & Vijg, 1993). Spots as a result of, for example, contamination with dirt particles or other artefacts rarely occur and can easily be distinguished from genuine spots. It should be noted that among different DNA samples variation can occur in the total number of spots detected. In general, fewer spots are detected when more stringent hybridisation and washing conditions are applied, when smaller amounts (<5 μg) of genomic DNA are loaded on the gel or when DNA quality is low (extensively discussed in Uitterlinden & Vijg, 1993). For 2-D DNA typing the quality of the DNA isolated is more important than for the relatively simple standard Southern blot analysis. This is because single-strand breaks have an effect on the second-dimension separation process (Uitterlinden & Vijg, 1993). This can be assessed by alkaline agarose gel electrophoresis of undigested genomic DNA (Sambrook *et al.*, 1989). Upon such analysis, 10 out of 18 tumour/normal DNA samples used for the Southern blot analyses appeared to be suitable for 2-D DNA typing.

The tumour/WBC combinations were analysed with four different probes: 33.15, 33.6, $(CAC)_n$ and TELO. The first three probes generated multilocus hybridisation patterns containing approximately 300 spots, while TELO resulted in a simple spot pattern consisting of about 50 spots. On average, 863 spots were scored for all probes together per individual after correction for spot overlap. Because of the much higher resolution of 2-D DNA typing, the overlap detectable is much less than for Southern blot analysis (overlap between any of the four probes varies from 6 to 12%). Figure 2 shows two-dimensional spot patterns of tumour and normal WBC DNA from patient 43 after hybridisation with core probes 33.15 and TELO.

A considerable fraction of the spots observed in the 2-D DNA types generated with probes 33.15, 33.6 and $(CAC)_n$ is polymorphic, that is spot variants can be observed between different individuals. From the analysis of human reference pedigrees of the Centre d'Étude Polymorphisme Humain (CEPH; Paris, France) this fraction is estimated to be at least 80% (E. Mullaart *et al.*, manuscript in preparation). For TELO the polymorphic fraction is considerably lower.

On average we detected 18 differences per individual for the four probes together (range 7–37). In all patients differences were observed (on average 2.1%; range 1.1–5.2%) between tumour and normal (blood) tissue. The differences observed are summarised in Table III. Some details of the 2-D DNA typing patterns of the WBC and the

**Table II** Frequency of genetic changes in breast tumours as detected by Southern blot hybridisation analysis

| | | CAC | | | 33.15 | | | INS | | | 33.6 | | | TCC | | | HBV-1 | |
|---|---|---|---|---|---|---|---|---|---|---|---|---|---|---|---|---|---|---|
| Patient no. | Total | G/A | Loss | Total | G/A | Loss | Total | G/A | Loss | Total | G/A | Loss | Total | G/A | Loss | Total | G/A | Loss |
| 16 | 35 | 3 | 4 | 35 | 1 | 4 | 28 | 2 | 1 | 23 | | 1 | 20 | | | 24 | | 1 |
| 18 | 36 | | | 36 | | | 31 | | | 27 | | | 17 | | | 22 | | |
| 31 | 41 | 1 | 3 | 37 | | 3 | 32 | | 2 | 26 | | 1 | 21 | | 1 | 17 | | 1 |
| 32 | 34 | | | 33 | | | 25 | | | 25 | | | 13 | | | 13 | | |
| 33 | 31 | | 1 | 32 | | | 27 | | | 25 | | 1 | 19 | | | 18 | | |
| 38 | 34 | 1 | 2 | 37 | 2 | | 29 | | | 30 | | | 20 | | | 21 | | |
| 43 | 28 | 2 | | 30 | | 1 | 24 | | 1 | 21 | 1 | 1 | 22 | | 1 | 22 | | |
| 45 | 33 | | | 33 | | | 27 | | | 26 | | 2 | 20 | | | 18 | | |
| 51 | 35 | 1 | 3 | 34 | 1 | | 23 | 1 | | 17 | | 1 | 19 | | 1 | 12 | | |
| 54 | 35 | | | 31 | | | 28 | | | 25 | | | 25 | | | 26 | | |
| 57 | 31 | | 4 | 31 | 1 | 3 | 28 | 2 | 2 | 25 | | 3 | 24 | | 1 | 20 | | |
| 59 | 30 | | | 31 | | 1 | 26 | | | 27 | | 2 | 21 | | 2 | 15 | | 2 |
| 61 | 34 | | | 25 | | | 26 | 1 | | 20 | | 1 | 19 | | 1 | 13 | | |
| 65 | 35 | | | 32 | | | 21 | | | 30 | | | 22 | | | 20 | | |
| 67 | 20 | | | 17 | | | 19 | | | 6 | | | 9 | | | 0 | | |
| 70 | 26 | | 2 | 28 | | | 23 | | | 8 | | 1 | 9 | | | 2 | | |
| 71 | 27 | 1 | 1 | 30 | | | 21 | 1 | | 18 | | 2 | 10 | | | 5 | | |
| 78 | 28 | | 2 | 31 | | | 21 | 1 | | 19 | | 1 | 12 | | 1 | 4 | | |
| Total | 573 | 9 | 22 | 563 | 5 | 12 | 459 | 8 | 6 | 398 | 1 | 17 | 322 | 0 | 8 | 272 | 0 | 4 |
| Average | 32 | 0.5 | 1.2 | 31 | 0.3 | 0.7 | 26 | 0.4 | 0.3 | 22 | 0.1 | 0.9 | 18 | 0.0 | 0.4 | 15 | 0.0 | 0.2 |

| | | GATA | | | TELO | | | GACA | | | | |
|---|---|---|---|---|---|---|---|---|---|---|---|---|
| Patient no. | Total | G/A | Loss | Total | G/A | Loss | Total | G/A | Loss | Total G/A | Total Loss | Total bands |
| 16 | 20 | | | 7 | | | 9 | | | 5 | 8 | 201 |
| 18 | 17 | | | 6 | 1 | | 6 | | | 1 | 0 | 198 |
| 31 | 23 | | 1 | 7 | | | 5 | | | 1 | 9 | 209 |
| 32 | 1 | | | 6 | | 1 | 4 | | | 0 | 1 | 154 |
| 33 | 20 | | | 6 | | | 5 | | | 0 | 2 | 183 |
| 38 | 19 | | | 8 | 2 | | 5 | | | 5 | 2 | 203 |
| 43 | 15 | | | 0 | | | 10 | | | 1 | 6 | 172 |
| 45 | 13 | | | 8 | | | 6 | | | 0 | 2 | 184 |
| 51 | 12 | | | 9 | | | 5 | 1 | | 4 | 4 | 166 |
| 54 | 17 | | | 6 | | | 6 | | | 0 | 0 | 199 |
| 57 | 10 | | 1 | 5 | | | 7 | | | 3 | 11 | 181 |
| 59 | 15 | | | 5 | | | 6 | | | 0 | 7 | 176 |
| 61 | 17 | | | 5 | | | 6 | | 1 | 1 | 3 | 165 |
| 65 | 11 | | | 7 | | | 8 | | | 0 | 0 | 186 |
| 67 | 8 | | | 4 | | | 4 | | 1 | 0 | 1 | 87 |
| 70 | 10 | | | 6 | | | 5 | | | 0 | 3 | 117 |
| 71 | 12 | 1 | | 7 | | | 4 | | | 2 | 3 | 134 |
| 78 | 12 | | | 7 | | | 4 | | | 1 | 4 | 138 |
| Total | 252 | 1 | 2 | 109 | 3 | 1 | 105 | 1 | 2 | 24 | 66 | 3053 |
| Average | 14 | 0.1 | 0.1 | 6 | 0.2 | 0.1 | 6 | 0.1 | 0.1 | 1 | 4 | 170 |

Total number of bands corrected for overlap between different probes. G/A = gain and/or amplification.

**Figure 2** Two-dimensional DNA typing patterns of breast tumour DNA (tumour) and WBC DNA (normal) from patient 43 after hybridisation with core probes 33.15 and TELO. Squares indicate spot losses in tumour DNA and the corresponding spot position in normal DNA. UF, urea/formamide denaturant.

corresponding tumour tissue are shown in Figure 3. To assess the reproducibility of the spot differences found, each comparison between tumour and normal DNA was performed at least in duplicate. Table IV shows the results of these replicate experiments. Spot differences found in hybridisation patterns of one gel could be reproduced in about 50% of the cases in the corresponding patterns from another gel. Only these reproducible spot differences were used in Table III, which is therefore an underestimate of the real number of differences.

As in the Southern blot analysis, 74% of the differences appeared to be spot losses in the tumour, while 26% spot gains/amplifications were observed. Of the latter, 20% were amplifications, 5% involved gains and 1% were other types of differences such as shifts in spot position. Probes 33.15, 33.6 and TELO were more efficient in detecting spot differences than $(CAC)_n$, although TELO detected fewer spots than 33.15 and 33.6. Similar to what was found for the Southern blot analysis, probe 33.6 almost exclusively detected spot losses. From Table III it can be derived that the frequency of changes (percentage of tumour-specific spot variants) differed considerably from patient to patient. In this respect the results corroborate the Southern blot analyses, albeit a much larger number of detected loci substantiate the 2-D DNA typing results [i.e. a total number of 863 spots scored as compared with 91 bands (for the four probes) in the Southern blot analysis]. The variation among patients is illustrated by Figure 4, in which the genetic changes detected with core probes 33.6, 33.15, $(CAC)_n$, and TELO by Southern analysis were combined with those obtained by 2-D DNA typing with the same core probes (for the 1-D results 20% overlap was used to correct for the total number of bands detected). The figure indicates that the micro- and minisatellite core probes used have the same tendency but detect different spectra among patients with respect to the frequency of changes detected in a given patient. By compar-

**Table III** Frequency of genetic changes in breast tumours as detected by 2-D typing

| Patient no. | 33.6 Total | G/A | % | Loss | % | G/A + L | % | 33.15 Total | G/A | % | Loss | % | G/A + L | % | CAC Total | G/A | % | Loss | % | G/A + L | % |
|---|---|---|---|---|---|---|---|---|---|---|---|---|---|---|---|---|---|---|---|---|---|
| 38 | 219 | 0 | 0.0 | 3 | 1.4 | 3 | 1.4 | 265 | 0 | 0.0 | 8 | 3.0 | 8 | 3.0 | 362 | 0 | 0.0 | 6 | 1.7 | 6 | 1.7 |
| 43 | 243 | 0 | 0.0 | 5 | 2.1 | 5 | 2.1 | 378 | 0 | 0.0 | 3 | 0.8 | 3 | 0.8 | 484 | 0 | 0.0 | 8 | 1.7 | 8 | 1.7 |
| 45 | 302 | 0 | 0.0 | 6 | 2.0 | 6 | 2.0 | 309 | 0 | 0.0 | 5 | 1.6 | 5 | 1.6 | 364 | 1 | 0.3 | 14 | 3.8 | 15 | 4.1 |
| 51 | 215 | 0 | 0.0 | 0 | 0.0 | 0 | 0.0 | 359 | 1 | 0.3 | 2 | 0.6 | 3 | 0.8 | 352 | 0 | 0.0 | 7 | 2.0 | 7 | 2.0 |
| 54 | 317 | 0 | 0.0 | 2 | 0.6 | 2 | 0.6 | 371 | 1 | 0.3 | 2 | 0.5 | 3 | 0.8 | 205 | 0 | 0.0 | 7 | 3.4 | 7 | 3.4 |
| 57 | 293 | 0 | 0.0 | 0 | 0.0 | 0 | 0.0 | 314 | 4 | 1.3 | 7 | 2.2 | 11 | 3.5 | 348 | 6 | 1.7 | 7 | 2.0 | 13 | 3.7 |
| 59 | 182 | 2 | 1.1 | 8 | 4.4 | 10 | 5.5 | 214 | 5 | 2.3 | 3 | 1.4 | 8 | 3.7 | 325 | 7 | 2.2 | 6 | 1.8 | 13 | 4.0 |
| 61 | 176 | 0 | 0.0 | 0 | 0.0 | 0 | 0.0 | 125 | 1 | 0.8 | 0 | 0.0 | 1 | 0.8 | 339 | 2 | 0.6 | 1 | 0.3 | 3 | 0.9 |
| 71 | 138 | 0 | 0.0 | 2 | 1.4 | 2 | 1.4 | 232 | 0 | 0.0 | 0 | 0.0 | 0 | 0.0 | 395 | 6 | 1.5 | 1 | 0.3 | 7 | 1.8 |
| 78 | 228 | 4 | 1.8 | 2 | 0.9 | 6 | 2.6 | 222 | 0 | 0.0 | 0 | 0.0 | 0 | 0.0 | 495 | 4 | 0.8 | 4 | 0.8 | 8 | 1.6 |
| Total | 2313 | 6 | | 28 | | 34 | | 2789 | 12 | | 30 | | 42 | | 3669 | 26 | | 61 | | 87 | |
| Average | 231 | 0.6 | 0.3 | 2.8 | 1.3 | 3.4 | 1.6 | 279 | 1.2 | 0.5 | 3.0 | 1.0 | 4.2 | 1.5 | 367 | 2.6 | 0.7 | 6.1 | 1.8 | 8.7 | 2.5 |

| Patient no. | TELO Total | G/A | % | Loss | % | G/A + L | % | Total Total | G/A | % | Loss | % | G/A + L | % |
|---|---|---|---|---|---|---|---|---|---|---|---|---|---|---|
| 38 | 60 | 0 | 0.0 | 0 | 0.0 | 0 | 0.0 | 843 | 0 | 0.0 | 17 | 2.0 | 17 | 2.0 |
| 43 | 49 | 0 | 0.0 | 3 | 6.1 | 3 | 4.1 | 1074 | 0 | 0.0 | 19 | 1.8 | 19 | 1.8 |
| 45 | 53 | 0 | 0.0 | 1 | 1.9 | 1 | 1.9 | 956 | 1 | 0.1 | 26 | 2.7 | 27 | 2.8 |
| 51 | 53 | 0 | 0.0 | 1 | 1.9 | 1 | 1.9 | 911 | 1 | 0.1 | 10 | 1.1 | 11 | 1.2 |
| 54 | 53 | 0 | 0.0 | 1 | 1.9 | 1 | 1.9 | 880 | 1 | 0.1 | 12 | 1.4 | 13 | 1.5 |
| 57 | 50 | 1 | 2.0 | 2 | 4.0 | 3 | 6.0 | 935 | 11 | 1.2 | 16 | 1.7 | 27 | 2.9 |
| 59 | 50 | 2 | 4.0 | 4 | 8.0 | 6 | 12.0 | 717 | 16 | 2.2 | 21 | 2.9 | 37 | 5.2 |
| 61 | 50 | 1 | 2.0 | 2 | 4.0 | 3 | 6.0 | 642 | 4 | 0.6 | 3 | 0.5 | 7 | 1.1 |
| 71 | 52 | 0 | 0.0 | 0 | 0.0 | 0 | 0.0 | 760 | 6 | 0.8 | 3 | 0.4 | 9 | 1.2 |
| 78 | 34 | 0 | 0.0 | 3 | 8.8 | 3 | 8.8 | 911 | 8 | 0.9 | 9 | 1.0 | 17 | 1.9 |
| Total | 504 | 4 | | 17 | | 21 | | 8629 | 48 | | 136 | | 184 | |
| Average | 50 | 0.4 | 0.8 | 1.7 | 3.7 | 2.1 | 4.3 | 863 | 4.8 | 0.6 | 13.6 | 1.5 | 18.4 | 2.1 |

The total number of spots detected is corrected for overlap between the different probes. G/A = Gain and/or amplification. L, loss.

**Figure 3** Details taken from 2-D DNA typing patterns of normal versus tumour DNAs from three different patients after hybridisation with probe 33.6. Squares indicate spot losses, arrows indicate shifts in position.

**Table IV** Reproducibility of spot differences[a]

| *Patient no.* | *Gel no.* | *Total no. of diff.*[b] | *No. of reproducible diff.* | *Percentage* |
|---|---|---|---|---|
| 38 | 188 | 11 | 5 | 45 |
| | 150 | 14 | 5 | 36 |
| 43 | 145 | 14 | 11 | 79 |
| | 161 | 13 | 11 | 85 |
| 45 | 183 | 11 | 6 | 55 |
| | 162 | 15 | 6 | 40 |
| 51 | 184 | 9 | 5 | 56 |
| | 62 | 6 | 5 | 83 |
| 54 | 172 | 11 | 6 | 55 |
| | 185 | 14 | 6 | 43 |
| 57 | 158 | 18 | 8 | 44 |
| | 175 | 14 | 8 | 57 |
| 59 | 199 | 10 | 6 | 60 |
| | 159 | 12 | 6 | 50 |
| | 200 | 6 | 6 | 100 |
| 61 | 151 | 6 | 1 | 17 |
| | 176 | 1 | 1 | 100 |
| 65 | 177 | 6 | 2 | 33 |
| | 170 | 3 | 2 | 67 |
| 71 | 144 | 19 | 9 | 47 |
| | 168 | 12 | 9 | 75 |
| 78 | 140 | 5 | 3 | 60 |
| | 178 | 11 | 3 | 27 |
| | | | Average | 59 |

[a]Detected by probe 33.15. [b]Total number of differences detected between WBC DNA and tumour DNA.

ing 2-D DNA typing patterns for each core probe for different patients we could detect differences occurring in more than one patient. Figure 5 shows the standardised 2-D gel positions of all such differences.

**Figure 4** Percentage of genetic changes of the total number of fragments detected by both 2-D DNA typing and Southern blot hybridisation analysis, in breast tumours of different patients using each of four different core probes [33.6 (■), 33.15 (□), $(CAC)_n$ (◆), and TELO (◇)]. Sample 78 is a local recurrence.

In view of the small number of individual tumours analysed no attempt was made to test for significant associations between spot changes and node status or recurrence.

### Discussion

In the present paper we demonstrate the potenty of 2-D DNA typing in genomic scanning of primary breast tumours. In each breast cancer analysed at least four genomic changes

**Figure 5** Standardised positions in the 2-D DNA typing patterns of tumour-specific spot variants detected in at least two different patients using four different core probes [33.15, 33.6, $(CAC)_n$, and TELO]. For each core probe used the standardised grid and the constant spots used to compare different gels are shown. Circles indicate gains/amplifications. Squares indicate spot losses. Numbers adjacent to the circles/squares indicate the patient number.

could be observed, making this approach the most informative genome scanning technique currently available. The total number of 863 spots detected should correspond to at least half this number of loci, assuming 100% heterozygosity, no overlap and no *Hae*III-sensitive sites in the detected alleles. In segregation studies using the CEPH panel of multigenerational pedigrees we have established that about 80% of the spots for a given probe (probe 33.6) behave in a Mendelian fashion and thus correspond to alleles of polymorphic loci (te Meerman *et al.*, 1993; Mullaart *et al.*, manuscript in preparation).

The high-resolution genome coverage can easily be extended by sequential rehybridisations with additional probes. The 2-D system is therefore much more powerful than Southern analysis in assessing the amount of genetic change in tumours. It should be noted that because of the different size range other DNA fragments are detected in this 2-D approach than in the standard Southerns; in the latter, agarose-based system, only the large alleles can be scored. In this sense the 1-D and 2-D approaches are complementary. To improve the efficiency of 2-D DNA typing in scoring polymorphic loci even further, the system can be coupled to automated image analysis and database programs. Such programs, which are commercially available, are presently being tested for their efficiency in rapidly interpreting 2-D DNA typing patterns.

From this study it appears that the majority (74%) of genetic changes detected involve deletions of bands/spots, whereas amplifications represent only 20% and gains 5%. In this respect genome scanning by 2-D DNA typing using micro- and minisatellite core probes provides a more accurate representation of genomic alteration in tumour genomes than alternative approaches such as RLGS, in which only amplifications can be detected (Hirotsune *et al.*, 1992). We are currently extending the 2-D DNA typing analysis by including more tumour samples from the same series of patients (Andersen *et al.*, 1992). Since these tumours have also been genotyped for a number of loci, this allows the results on allelic imbalance obtained by 2-D DNA typing to be compared with those obtained using locus-specific markers.

New spots (gains) are most likely due to somatic mutations at the loci detected by the micro- and minisatellite core probes used. These loci include the hypervariable VNTR loci, mutations in which could arise through a number of mechanisms (Thein *et al.*, 1987; Armour *et al.*, 1989). Such mutations do not necessarily occur in the tumour during development and progression, but could also reflect mutations pre-existing in the normal cells from which the tumour arose.

To circumvent the lack of immediate information on the locus involved in a particular change, it is possible to obtain spots of interest by direct isolation from the gel. A simple and rapid procedure to accomplish this has recently been developed (W.J.F. de Leeuw *et al.*, submitted). Therefore, large-scale 2-D DNA typing of tumours allows the rapid development of large series of probes specific for particular genomic changes in a given cancer. Alternatively, spots can be identified on the basis of co-segregation with known marker alleles, for example by using the CEPH panel. Preliminary results indicate that this is feasible and a 2-D DNA typing genetic map is presently under construction (Mullaart *et al.*, manuscript in preparation). The availability of such a map will allow direct identification of variant spots in tumour tissue. Probes in the chromosomal region identified by the spots of interest can then be obtained from genomic libraries.

Using 2-D DNA typing the degree of genetic instability in a particular neoplasm can be assessed by scanning the total genome at high resolution. The higher genetic lability of tumours as compared with normal tissues is well documented (Nowell, 1976; Volpe, 1988). It should be noted that any distinction among tumour genomes with respect to genetic variability might be due to differences in (clonal) heterogeneity of the tumour tissue. Multiclonal tumours in which each clone will differ at multiple loci will result in a 2-D DNA typing pattern with few consistent differences from normal. Breast cancer early stages may be heterogeneous and of polyclonal nature before convergence to monoclonality takes place (Borg, 1992). It is not inconceivable that such an evolutionary process is reflected in the patterns of genetic changes we observed. However, any definite conclusions can only be drawn when results on many more samples become available.

The results obtained in this present study revealed a number of specific changes (amplifications and deletions) in breast cancers which had occurred in more than one patient. These spots correspond to alleles these patients happen to share in their normal DNA and which display allelic imbalance in the tumour. Such spots are therefore likely to be derived from loci more or less frequently involved in tumorigenesis and/or tumour progression. In this respect it should be noted that spot variants found to be on the same isotherm in the denaturing gradient (second dimension) could be alleles from the same locus (Uitterlinden & Vijg, 1991; Hovig *et al.*, 1993). These (polymorphic) spot variants (Figure 5) are prime candidates for follow-up studies. Indeed, some of these spots have been isolated directly from the gel and are presently being tested as locus-specific probes on larger numbers of primary breast tumours for their suitability as prognostic indicators. The polymorphic nature of these probes allows their use in studying genetic susceptibility of breast cancer. Finally, the availability of ample genetic markers will greatly facilitate the identification and isolation of genes determining tumour behaviour and susceptibility.

We thank Dr T.A.W. Splinter (Department of Oncology, Academic Hospital, Rotterdam) for critically reading the manuscript and Toyobo Ltd. Co. for financially supporting this study.

### References

ANDERSEN, T.I., GAUSTAD, A., OTTERSTAD, L., FARRANTS, G.W., NESLAND, J.M. TREIT, K.M. & BORRESEN, A.L. (1992). Genetic alterations of the tumour suppressor gene regions 3p, 11p, 13q, 17p and 17q in human breast carcinomas. *Genes. Chrom. Cancer*, **4**, 113–121.

ARMOUR, J.A.L., PATEL, I., THEIN, S.L., FEY, M.F. & JEFFREYS, A.J. (1989). Analysis of somatic mutations at human minisatellite loci in tumours and cell lines. *Genomics*, **4**, 328–334.

BORG, Å. (1992). *Gene Alterations in Human Breast Cancer* (thesis). University of Lund, Sweden.

FISCHER, S.G. & LERMAN, L.S. (1979). Length-independent separation of DNA restriction fragments in two-dimensional gel electrophoresis. *Cell*, **16**, 191–200.

HIROTSUNE, S., HATADA, I., KOMATSUBARA, H., NAGAI, H., KUMA, K., KOBAYAKAWA, K., KAWARA, T., NAKAGAWARA, A., FUJII, K., MUKAI, T. & HAYASHIZAKI, Y. (1992). New approach for detection of amplification in cancer DNA using restriction landmark genomic scanning. *Cancer Res.*, **52**, 3642–3647.

HOVIG, E., MULLAART, E., BÖRRESEN, A.-L., UITTERLINDEN, A.G. & VIJG, J. (1993). Genome scanning of human breast carcinomas using micro- and minisatellite core probes. *Genomics*, **17**, 66–75.

KUNKEL, L.M., SMITH, K.D., BOYER, S.H., BORGAONKAR, D.S., WACHTEL, S.S., MILLER, O.J., BREG, W.R., JONES Jr, H.W. & RARY, J.M. (1977). Analysis of human Y-chromsome-specific reiterated DNA in chromosome variants. *Proc. Natl Acad. Sci. USA*, **74**, 1245–1249.

TE MEERMAN, G.J., MULLAART, E., VAN DER MEULEN, M.A., DEN DAAS, J.H.G., MORROLLI, B., UITTERLINDEN, A.G. & VIJG, J. (1993). Linkage analysis by two-dimensional DNA typing. *Am. J. Hum. Genet.* (in press).

NOWELL, P. (1976). The clonal evolution of tumour cell populations. *Science*, **194**, 23–28.

PETO, R., ROE, F.J.C., LEE, P.N., LEVY, L. & CLACK, J. (1975). Cancer and ageing in mice and man. *Br. J. Cancer*, **32**, 411–426.

RUSSELL, S.J. (1992). The clinical applications of oncogene research. In *Introduction to the Molecular Genetics of Cancer*, Vile, R.G. (ed.) pp. 177–201. John Wiley: Chichester.

SAMBROOK, J., FRITSCH, E.F. & MANIATIS, T. (1989). *Molecular Cloning: A Laboratory Manual*, 2nd ed. Cold Spring Harbor Laboratory Press: Cold Spring Harbor, NY.

THEIN, S.L., JEFFREYS, A.J., GOOI, H.C., COTTER, F., FLINT, J., O'CONNOR, N.T.J., WEATHERALL, D.J. & WAINSCOAT, J.S. (1987). Detection of somatic changes in human cancer DNA by DNA fingerprint analysis. *Br. J. Cancer*, **55**, 353–356.

UITTERLINDEN, A.G. & VIJG, J. (1993). *Two-Dimensional DNA Typing: A Parallel Approach to Genome Analysis*. Horwood: Chichester (in press).

UITTERLINDEN, A.G., SLAGBOOM, P., KNOOK, D.L. & VIJG, J. (1989). Two-dimensional DNA fingerprinting of human individuals. *Proc. Natl Acad. Sci. USA*, **86**, 2742–2746.

UITTERLINDEN, A.G. & VIJG, J. (1991). Locus-specific electrophoretic migration patterns of minisatellite alleles in denaturing gradient gels. *Electrophoresis*, **12**, 12–16.

VOLPE, J.P.G. (1988). Genetic instability of cancer: why a metastatic tumour is unstable and a benign tumour is stable. *Cancer Genet. Cytogenet.*, **34**, 125–134.